# Crystal structure of the bora­benzene–2,6-lutidine adduct

**DOI:** 10.1107/S2056989015020599

**Published:** 2015-11-14

**Authors:** Lauri Kivijärvi, Matti Haukka

**Affiliations:** a Department of Chemistry, University of Jyväskylä, PO Box 35, FI-40014 Jyväskylä, Finland

**Keywords:** crystal structure, bora­benzene, π–π stacking

## Abstract

In the title compound, C_12_H_14_BN, the complete mol­ecule is generated by a crystallographic twofold axis, with two C atoms, the B atom and the N atom lying on the rotation axis. The dihedral angle between the bora­benzene and pyridine rings is 81.20 (6)°. As well as dative electron donation from the N atom to the B atom [B—N = 1.5659 (18) Å], the methyl substituents on the lutidine ring shield the B atom, which further stabilizes the mol­ecule. In the crystal, weak aromatic π–π stacking between the pyridine rings [centroid–centroid separation = 3.6268 (9) Å] is observed, which generates [001] columns of mol­ecules.

## Related literature   

For the synthesis of the title compound, see: Hoic *et al.* (1996 ▸). For a related structure, see: Boese *et al.* (1985 ▸). For bora­benzene adducts as analogues of cyclo­penta­dienyl anions (Cp), see: Bazan *et al.* (2000 ▸); Wang *et al.* (2002 ▸); Cui *et al.* (2010 ▸). For the uses of bora­benzenes and their metal complexes, see: Wang *et al.* (2002 ▸; Jaska *et al.* (2006 ▸).
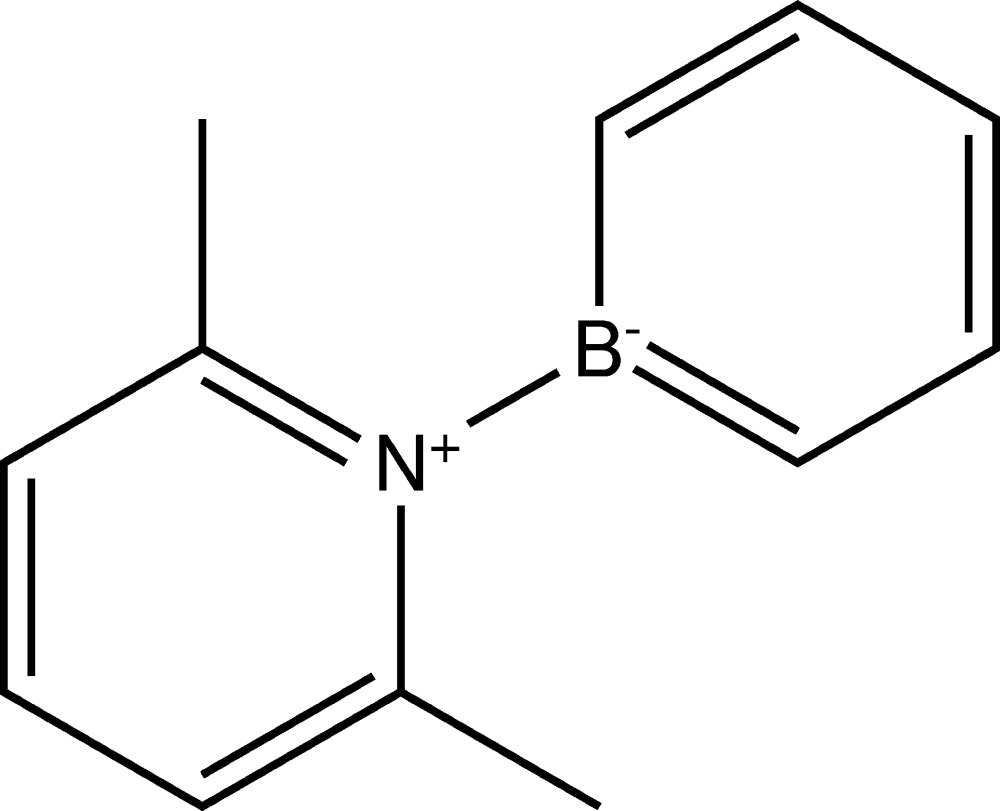


## Experimental   

### Crystal data  

C_12_H_14_BN
*M* *_r_* = 183.05
Monoclinic, 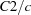

*a* = 10.008 (2) Å
*b* = 14.447 (3) Å
*c* = 7.1360 (14) Å
β = 90.16 (3)°
*V* = 1031.8 (4) Å^3^
*Z* = 4
Mo *K*α radiation
μ = 0.07 mm^−1^
*T* = 120 K
0.24 × 0.18 × 0.16 mm

### Data collection  

Bruker Kappa APEXII CCD diffractometer
Absorption correction: multi-scan (*SADABS*; Bruker, 2012 ▸) *T*_min_ = 0.646, *T*_max_ = 0.746
7258 measured reflections
1482 independent reflections
1280 reflections with *I* > 2σ(*I*)
*R*_int_ = 0.028

### Refinement  

*R*[*F* ^2^ > 2σ(*F* ^2^)] = 0.044
*wR*(*F* ^2^) = 0.120
*S* = 1.06
1482 reflections
67 parameters
H-atom parameters constrained
Δρ_max_ = 0.30 e Å^−3^
Δρ_min_ = −0.17 e Å^−3^

### 

Data collection: *COLLECT* (Bruker, 2008 ▸); cell refinement: *DENZO*/*SCALEPACK* (Otwinowski & Minor, 1997 ▸); data reduction: *DENZO*/*SCALEPACK*; program(s) used to solve structure: *SUPERFLIP* (Palatinus & Chapuis, 2007 ▸); program(s) used to refine structure: *SHELXL2014* (Sheldrick, 2015 ▸); molecular graphics: *CHIMERA* (Pettersen *et al.*, 2004 ▸); software used to prepare material for publication: *SHELXL2014*.

## Supplementary Material

Crystal structure: contains datablock(s) I. DOI: 10.1107/S2056989015020599/hb7526sup1.cif

Structure factors: contains datablock(s) I. DOI: 10.1107/S2056989015020599/hb7526Isup2.hkl

Supporting information file. DOI: 10.1107/S2056989015020599/hb7526Isup3.mol

Supporting information file. DOI: 10.1107/S2056989015020599/hb7526Isup4.cml

**Fig. 1.**
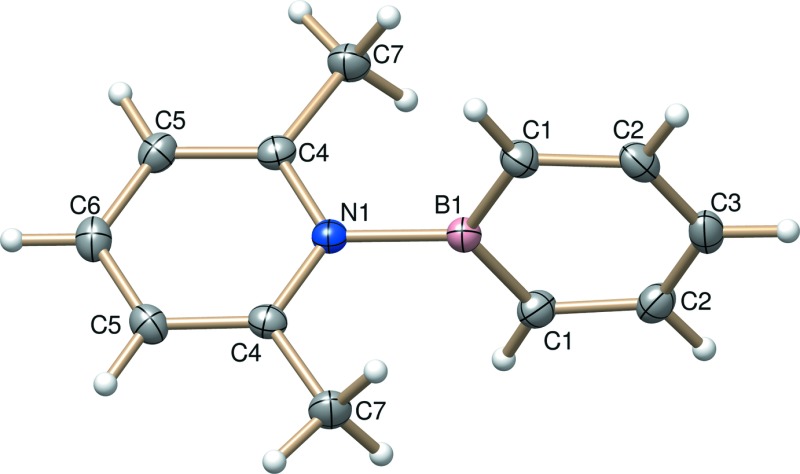
The molecular structure of the title compound, with atom labels and 50% probability displacement ellipsoids for non-H atoms.

. DOI: 10.1107/S2056989015020599/hb7526fig1.tif
The mol­ecular structure of the title compound, with atom labels and 50% probability displacement ellipsoids for non-H atoms.

CCDC reference: 1434350

Additional supporting information:  crystallographic information; 3D view; checkCIF report

## supplementary crystallographic information

### S1. Structural commentary

The title compound lies on a two-fold rotational axis, which passes through atoms H3, C3, B1, N1, C6, and H6.

Bora­benzene-2,6-lutidine is an example of nitro­gen-stabilized bora­benzene adducts. The nitro­gen atom of the base (2,5-lutidine) donates an electron pair to the boron atom, and thus stabilizes the bora­benzene ring. Bora­benzene-2,6-lutidine has a zwitterionic nature. The nitro­gen ring bears a positive charge, and the boron ring a negative charge.

Bora­benzenes are analogous to cyclo­penta­dienyl anions (Cp), althought they are generally weaker electron donors than Cp (Bazan *et al.*, 2000; Wang *et al.*, 2002 and Cui *et al.*, 2010). Bora­benzene rings can thus be used as a replacement for Cp when weaker electron donation properties are required. There is a growing inter­est to utilize bora­benzenes and their metal complexes in several applications including catalytic and semiconducting materials as well as light-emitting devices (Wang *et al.*, 2002 and Jaska *et al.*, 2006).

### S2. Synthesis and crystallization

The compound was synthesized according to the previously reported procedure (Hoic *et al.* 1996). X-ray quality crystals were obtained by using the following procedure: In a glove box, bora­benzene-2,6-lutidine was dissolved in pure toluene at room temperature until a saturated solution was obtained. The clear solution was separated and and the solution was allowed to evaporate slowly. Formed crystals were collected from the solution after one week and were immediately taken to an X-ray diffraction analysis. In order to protect the crystals from air and moisture, the crystals were immersed to cryo oil before taking them out from the glove box.

### S3. Refinement

Crystal data, data collection and structure refinement details are summarized in Table 1. Hydrogen atoms were positioned geometrically and constrained to ride on their parent atoms, with C—H = 0.95-0.98 Å and U_iso_ = 1.2-1.5 U_eq_(parent atom). The highest peak is located 0.69 Å from atom C5 and the deepest hole is located 0.67 Å from atom N1.

### Crystal data

**Table:**

C_12_H_14_BN	*F*(000) = 392
*M**_r_* = 183.05	*D*_x_ = 1.178 Mg m^−^^3^
Monoclinic, *C*2/*c*	Mo *K*α radiation, λ = 0.71073 Å
*a* = 10.008 (2) Å	Cell parameters from 10098 reflections
*b* = 14.447 (3) Å	θ = 1.0–28.7°
*c* = 7.1360 (14) Å	µ = 0.07 mm^−^^1^
β = 90.16 (3)°	*T* = 120 K
*V* = 1031.8 (4) Å^3^	Needle, yellow
*Z* = 4	0.24 × 0.18 × 0.16 mm

### Data collection

**Table:**

Bruker Kappa APEXII CCD diffractometer	1482 independent reflections
Radiation source: fine-focus sealed tube	1280 reflections with *I* > 2σ(*I*)
Horizontally mounted graphite crystal monochromator	*R*_int_ = 0.028
Detector resolution: 16 pixels mm^-1^	θ_max_ = 30.0°, θ_min_ = 4.0°
φ scans and ω scans with κ offset	*h* = −14→14
Absorption correction: multi-scan (*SADABS*; Bruker, 2012)	*k* = −16→20
*T*_min_ = 0.646, *T*_max_ = 0.746	*l* = −9→9
7258 measured reflections	

### Refinement

**Table:**

Refinement on *F*^2^	0 restraints
Least-squares matrix: full	Hydrogen site location: inferred from neighbouring sites
*R*[*F*^2^ > 2σ(*F*^2^)] = 0.044	H-atom parameters constrained
*wR*(*F*^2^) = 0.120	*w* = 1/[σ^2^(*F*_o_^2^) + (0.0555*P*)^2^ + 0.6142*P*] where *P* = (*F*_o_^2^ + 2*F*_c_^2^)/3
*S* = 1.06	(Δ/σ)_max_ < 0.001
1482 reflections	Δρ_max_ = 0.30 e Å^−^^3^
67 parameters	Δρ_min_ = −0.17 e Å^−^^3^

### Special details

**Table:**

Geometry. All e.s.d.'s (except the e.s.d. in the dihedral angle between two l.s. planes) are estimated using the full covariance matrix. The cell e.s.d.'s are taken into account individually in the estimation of e.s.d.'s in distances, angles and torsion angles; correlations between e.s.d.'s in cell parameters are only used when they are defined by crystal symmetry. An approximate (isotropic) treatment of cell e.s.d.'s is used for estimating e.s.d.'s involving l.s. planes.

### Fractional atomic coordinates and isotropic or equivalent isotropic displacement parameters (Å^2^)

**Table:**

	*x*	*y*	*z*	*U*_iso_*/*U*_eq_	
N1	0.5000	0.38254 (7)	0.7500	0.0182 (2)	
C1	0.43941 (9)	0.22210 (7)	0.59114 (13)	0.0229 (2)	
H1	0.3993	0.2534	0.4883	0.028*	
C2	0.44362 (10)	0.12556 (7)	0.59879 (14)	0.0246 (2)	
H2	0.4069	0.0910	0.4977	0.030*	
C3	0.5000	0.07806 (9)	0.7500	0.0254 (3)	
H3	0.5000	0.0123	0.7500	0.031*	
C4	0.38816 (9)	0.42921 (7)	0.80474 (12)	0.0197 (2)	
C5	0.38693 (9)	0.52523 (7)	0.80370 (13)	0.0225 (2)	
H5	0.3086	0.5578	0.8398	0.027*	
C6	0.5000	0.57352 (9)	0.7500	0.0241 (3)	
H6	0.5000	0.6393	0.7500	0.029*	
C7	0.26941 (10)	0.37460 (7)	0.86747 (15)	0.0258 (2)	
H7A	0.2957	0.3332	0.9699	0.039*	
H7B	0.2351	0.3379	0.7624	0.039*	
H7C	0.1996	0.4169	0.9112	0.039*	
B1	0.5000	0.27415 (10)	0.7500	0.0195 (3)	

### Atomic displacement parameters (Å^2^)

**Table:**

	*U*^11^	*U*^22^	*U*^33^	*U*^12^	*U*^13^	*U*^23^
N1	0.0180 (5)	0.0187 (5)	0.0179 (5)	0.000	−0.0021 (4)	0.000
C1	0.0222 (4)	0.0228 (5)	0.0239 (5)	−0.0022 (3)	−0.0018 (3)	0.0003 (3)
C2	0.0222 (4)	0.0235 (5)	0.0282 (5)	−0.0047 (3)	0.0024 (4)	−0.0045 (4)
C3	0.0230 (6)	0.0179 (6)	0.0355 (7)	0.000	0.0068 (5)	0.000
C4	0.0183 (4)	0.0224 (4)	0.0183 (4)	0.0011 (3)	−0.0013 (3)	0.0003 (3)
C5	0.0237 (5)	0.0221 (5)	0.0218 (5)	0.0042 (3)	0.0006 (3)	−0.0004 (3)
C6	0.0309 (7)	0.0193 (6)	0.0220 (6)	0.000	−0.0004 (5)	0.000
C7	0.0195 (4)	0.0268 (5)	0.0312 (5)	−0.0010 (3)	0.0025 (4)	−0.0003 (4)
B1	0.0177 (6)	0.0177 (6)	0.0230 (6)	0.000	−0.0002 (5)	0.000

### Geometric parameters (Å, º)

**Table:**

N1—C4^i^	1.3647 (11)	C4—C5	1.3874 (13)
N1—C4	1.3647 (11)	C4—C7	1.4961 (13)
N1—B1	1.5659 (18)	C5—C6	1.3843 (12)
C1—C2	1.3964 (14)	C5—H5	0.9500
C1—B1	1.4881 (12)	C6—C5^i^	1.3843 (12)
C1—H1	0.9500	C6—H6	0.9500
C2—C3	1.3965 (13)	C7—H7A	0.9800
C2—H2	0.9500	C7—H7B	0.9800
C3—C2^i^	1.3966 (13)	C7—H7C	0.9800
C3—H3	0.9500	B1—C1^i^	1.4881 (12)
			
C4^i^—N1—C4	120.78 (11)	C6—C5—C4	119.88 (9)
C4^i^—N1—B1	119.61 (6)	C6—C5—H5	120.1
C4—N1—B1	119.61 (6)	C4—C5—H5	120.1
C2—C1—B1	117.56 (9)	C5^i^—C6—C5	119.48 (13)
C2—C1—H1	121.2	C5^i^—C6—H6	120.3
B1—C1—H1	121.2	C5—C6—H6	120.3
C1—C2—C3	122.22 (9)	C4—C7—H7A	109.5
C1—C2—H2	118.9	C4—C7—H7B	109.5
C3—C2—H2	118.9	H7A—C7—H7B	109.5
C2—C3—C2^i^	121.13 (13)	C4—C7—H7C	109.5
C2—C3—H3	119.4	H7A—C7—H7C	109.5
C2^i^—C3—H3	119.4	H7B—C7—H7C	109.5
N1—C4—C5	119.99 (9)	C1—B1—C1^i^	119.30 (12)
N1—C4—C7	118.55 (9)	C1—B1—N1	120.35 (6)
C5—C4—C7	121.46 (8)	C1^i^—B1—N1	120.35 (6)
			
B1—C1—C2—C3	1.10 (12)	C4—C5—C6—C5^i^	0.52 (6)
C1—C2—C3—C2^i^	−0.59 (7)	C2—C1—B1—C1^i^	−0.53 (6)
C4^i^—N1—C4—C5	0.52 (6)	C2—C1—B1—N1	179.47 (6)
B1—N1—C4—C5	−179.47 (6)	C4^i^—N1—B1—C1	−98.80 (6)
C4^i^—N1—C4—C7	−178.65 (9)	C4—N1—B1—C1	81.20 (6)
B1—N1—C4—C7	1.35 (9)	C4^i^—N1—B1—C1^i^	81.20 (6)
N1—C4—C5—C6	−1.05 (12)	C4—N1—B1—C1^i^	−98.80 (6)
C7—C4—C5—C6	178.10 (7)		

Symmetry code: (i) −*x*+1, *y*, −*z*+3/2.
