# Supplementary figures and images for: Crystal structure of the bora­benzene–2,6-lutidine adduct
Source: Acta Crystallogr E Crystallogr Commun. 2015 Nov 14;71(Pt 12):o944. doi: 10.1107/S2056989015020599 (PMC4719914; doi:10.1107/S2056989015020599)

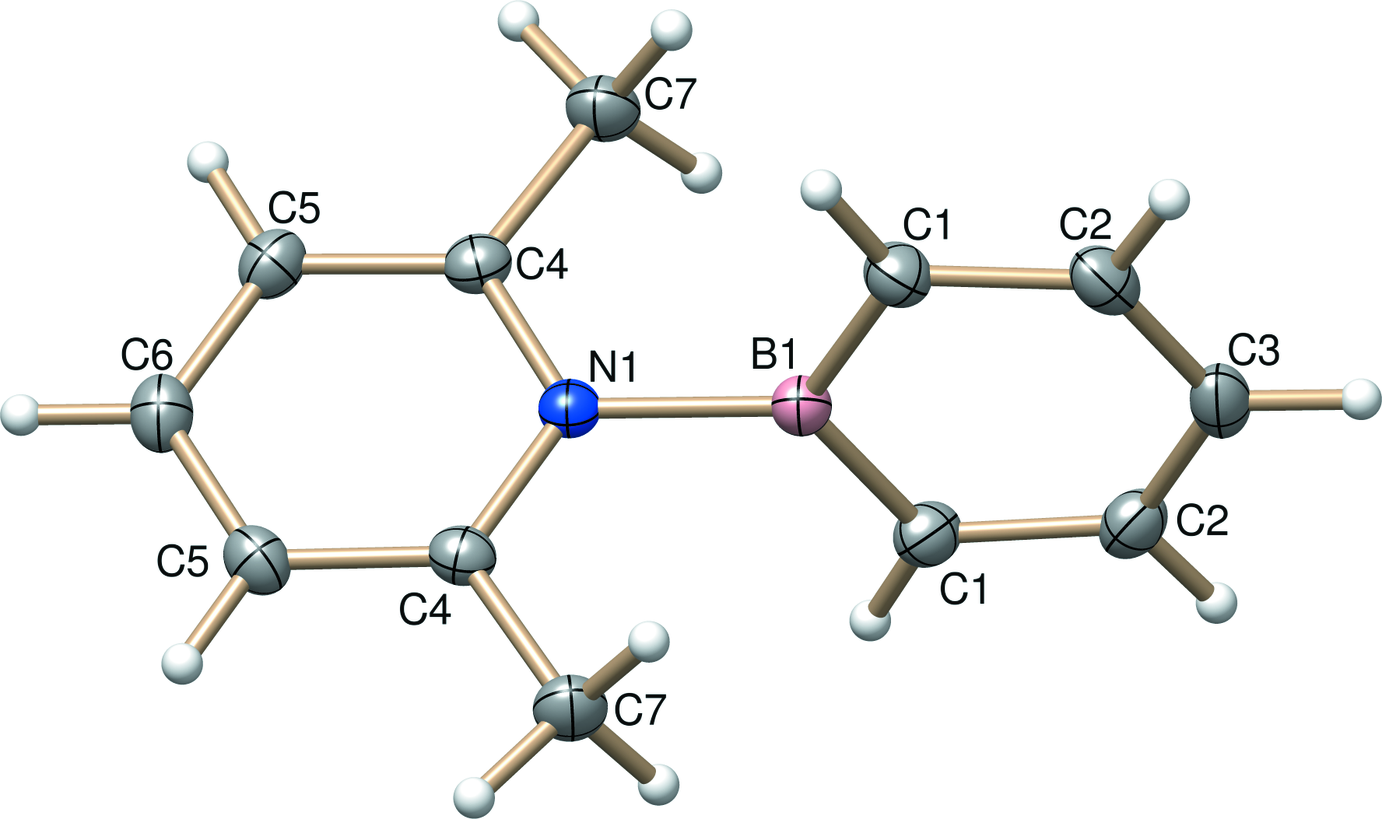

Supplement: Supplementary file 5 [file e-71-0o944-fig1.tif]
